# Antiseptic Therapeutics1Presented before the meeting of the American Veterinary Medical Association, Detroit, Mich., September, 1900.

**Published:** 1900-11

**Authors:** J. F. Winchester

**Affiliations:** Lawrence, Mass.


### THE JOURNAL

OF

### COMPARATIVE MEDICINE AND VETERINARY ARCHIVES.

VOL. XXI. *NOVEMBER, 1900.* NO. 11.

### ANTISEPTIC THERAPEUTICS.[1]

BY J. F. WINCHESTER, B.SC., D.V.S.,

LAWRENCE, MASS.

IN presenting this subject it is not necessary to introduce new matter, as new material is very likely to be misleading. Frequently theories are advanced, and accepted for a while, but in a short time are disproved. When old material is worked up with the view of showing that it was originally started on sound foundations, then a paper of this kind becomes useful. It becomes useful in another way, for if one can only say something calculated to create discussion and to encourage a free interchange of opinion, thus further good comes from it.

At the outset it is well to bear in mind what is meant by therapeutics and what an antiseptic is.

Therapeutics is that part of medicine which relates to the composition, the application, and the modes of operation of the remedies for diseases. It not only includes the administration of medicines, properly so-called, but also hygiene and dietetics or the application of diet and atmospheric and other non-medicinal influences to the preservation or recovery of health.

Dr. C. M. Sternberg tells us that by an antiseptic we mean an agent which prevents septic decomposition by preventing the development of the microörganisms which cause it, and the term is extended to the restraining influence exercised upon other microörganisms of the same class, including the pathogenic bacteria, or germs of infectious diseases. Miguel has formulated a table of the value of various antiseptics, the proportion being that in which the agents named permanently prevented the development of any

[1] Presented before the meeting of the American Veterinary Medical Association, Detroit, Mich., September, 1900.

of the microörganisms floating in the atmosphere—*i. e.*, the organic solution was freely exposed to the air and the test consisted in its permanent preservation from putrefactive changes. If Miguel had protected his solutions from atmospheric organism and had inoculated them with pure culture of pathogenic bacteria, the results would have been still more favorable, and after a time, which would be different for different bacteria, he would have found that the pathogenic bacteria introduced into his culture media would have lost their ability to grow in a favorable medium.

So, too, in therapeutics, if we can restrain the development of a pathogenic microörganism, we practically render it harmless, or at least restrict its power for mischief within limits which are compatible with the safety of the patient.

INDICATION. The term indication is used by the therapeutist as equivalent to "the pointing of nature for relief." The success of the physician depends on his skill in reading the indications. In many instances the indication is not for a single remedy, but for several, which, by acting together, will produce a combined result.

CAUSES. In studying the cause of an existing disorder for therapeutic purposes, it is first necessary to decide whether such cause has already ceased to act or whether it is still existent and persistent in its influence. When the cause has been a fleeting one, no indications exist in regard to it; when the cause persists, however, the indication is for the removal of the cause; in such a case are we most successful as therapeutists. When we disinfect a wound to destroy a malarial organism by quinine, we carry out the indication for the removal of the cause. The distribution of bacteria is universal. Wherever man, animals, or even plants live, die, and decompose, bacteria must be present. Notwithstanding their extreme familiarity with the animal body, there are certain parts of it into which bacteria do not enter, or entering remain vital for a very short time only, for the body juices and tissues of normal animals are free from them, and their occurrence in these places may almost always be accepted as a sign of disease.

Should the pabulum supplied to bacteria contain an excess of either alkali or acid, the growth of the organisms is inhibited. Most true bacteria grow best in a neutral or feebly alkaline medium. Light will usually attenuate the virulence of pathogenic bacteria. The presence of certain substances, especially some of the mineral salts, in an otherwise perfectly suitable medium, will prevent the development of bacteria, and when added to grown cultures of bacteria will destroy them.

Bacteria which produce diseases are known as pathogenic; those which do not, as non-pathogenic. Between the two groups there is no sharp line of separation, for true pathogens may be cultivated under such adverse conditions that their virulence will be entirely lost; while at times bacteria ordinarily harmless may be made toxic by certain manipulations or by introducing them into animals in certain combinations.

A microbe having effected its entrance into an animal may grow with such rapidity as to completely block the blood or lymph channels, thus stopping the proper circulation, and disease and death must result. More common than this is a local establishment of the organisms, with a resulting inflammation, due partly to the pressure of the foreign organisms and partly to their toxic metabolic products. The term pneumonia is a vague one, really comprehending a variety of inflammatory conditions of the lungs quite dissimilar in character. This being true, no one should be surprised to find that a single organism cannot be described as specific for all.

The bacterium which can be demonstrated in at least 75 per cent. of the cases of lobar pneumonia, and which is now almost universally accepted as the cause of the disease, and about whose specificity very few doubts can be raised, is the pneumococcus of Fränkel and Weichselbaum.

In catarrhal pneumonia the majority of the cases are caused by the presence of the staphylococcus and streptococcus of suppuration. It frequently happens that pneumonia occurs in the course of or shortly after the convalescence from influenza. In these cases both the influenza bacillus and the pneumococcus are present. Again, the pneumococcus and the staphylococci operate simultaneously and produce a purulent pneumonia with abscesses as the conspicuous feature. It was not until 1892, after the great epidemic of influenza, that there was found simultaneously by Canon and Pfeiffer a bacterium which conformed at least in large part to the requirements of specificity. It cannot be positively proven that this bacillus is the cause of influenza, but from the fact that the bacillus can be found only in cases of influenza, that its presence corresponds with the course of the disease in that it is present as long as the purulent secretions last, and then disappears, and that Pfeiffer was able to demonstrate its presence in all cases of uncomplicated influenza, it follows that his conclusion that the bacillus is specific is certainly justifiable.

The experiments of Delvine and Kole showed that the toxicity of the cultures resides not in a soluble toxin but in the bodies of the bacilli. The outcome of the researches was the total failure to produce immunity, and the subject is as susceptible as before, if not more so.

Recent researches in actinomycosis have changed the view held by some bacteriologists in regard to the actinomycoses, and have caused them to regard the organism as a bacillus. If it be a bacillus the central zone of granular cocci-like elements is to be regarded as consisting of individuals in process of rapid division and spore formation; the mycelial zone as consisting of perfect individuals, and the peripheral zone as consisting of individuals partly degenerated through the activity of the cells and tissue juices. The blood is known to have germicidal properties, and these properties have been studied by Von Fordor, who reaches some interesting conclusions. He finds that increased alkalescence of the blood raises its germicidal power very much. The drugs which do this most effectively are the carbonates of sodium and of potassium. By the alkalinization of the blood of rabbits he made a large percentage of them refractory to anthrax.

The alkali very frequently used by a great many is potash or some of its salts. The iodide of potassium closely resembles iodine, but is less powerful and devoid of local irritant action. It is an antiseptic. It promotes absorption of morbid products. Professors Thernassen and Nocard trust implicitly to the iodide in actinomycosis. In chronic poisoning with lead or mercury it removes the metals from the tissues and from the body.

Potassium permanganate, although it has not the antiseptic power of corrosive sublimate, effectually destroys bacteria. Goldberg, in summarizing the results obtained by all authors on the irrigation treatment of gonorrhœa, shows 60 per cent. cured within ten days, 40 per cent. within two weeks, while 5 per cent. required a longer period and 5 per cent. was recorded as failures. The success was brought about by the use of a solution of permanganate of potassium (ordinarily one part to three thousand parts of water), all forms of internal medication and nauseous dosing being done away with.

The volatile or essential oils are mostly of vegetable origin, being found generally in the flowers, leaves, fruit or seeds of plants, but they occur in all parts of the coniferæ. Most are found ready formed, but some, as the hydrocyanated almond and mustard oils, are produced by a species of fermentation. The chemical constitution of the volatile oils differs from that of the fixed oils; most are pure hydrocarbons, with the molecular formula of oil of turpentine ($C_{10}H_{16}$), and are termed *terpines.* The volatile oils are *antiseptics* and *stimulants.* Oil of turpentine in medicinal doses is an antiseptic and stimulant, especially of mucous and skin surfaces. Like other volatile oils, it is an active antiseptic. In destroying bacteria spores Koch found it more effective than alcohol, ether, chloroform, or benzol, as no spores germinated after being wet with it for five days. It is eliminated by the lungs; it acts as a stimulant, an antiseptic, and an expectorant, by the skin promoting diaphoresis; by the kidneys inducing diuresis. The camphors, physiologically as well as chemically, are volatile oils. In the form of a fine powder or a solution they are quickly absorbed; they stimulate the brain, spinal cord, heart, and respiratory functions, and are excreted by the skin and bronchial membrane, and also in smaller amount by the kidneys. They have considerable antiseptic power, Koch having found that 1 : 2500 parts of water hindered the development of anthrax bacilli.

The benzol benzene, or aromatic series of carbon compounds, includes a number of antiseptics and antipyretics, viz., benzene, carbolic acid, creolin, salicylic acid, salol, antipyrin, antifibrin, phenacetin, etc. Creolin has been proved by bacteriological tests to be more prompt and effective than carbolic acid in the destruction of the microbes of anthrax, fowl cholera, glanders, etc. Kaufmann states that as a bactericide it is *ten* times as powerful as carbolic acid, and that a 10 per cent. solution does not irritate the skin, even when abraded, nor the mucous membrane. It is inhaled in ozæna, laryngitis, pharyngitis, strangles, bronchitis, gangrenous pneumonia, and in purpura. In the form of an injection it is useful in the retention of placenta, metritis, and purulent cystitis.

The petroleums or paraffins are hydrocarbons produced by the decomposition of vegetable matter. They are obtained from the destructive distillation of coal, from the bituminous shales, and from the oil wells found in various parts of the world. The petroleum, distilling over between 300° and 400° F., is chiefly nonane ($C_9H_{20}$) and dodecane ($C_2H_{26}$), and is used for lubricant purposes. At higher temperatures there come off hexadecane ($C_{16}H_{34}$) and other paraffins richer in carbon, constituting such soft solids as vaseline and the soft petroleums; while still higher temperatures produce the hard paraffins. The petroleums belong physiologically to the fatty or alcohol series of hydrocarbons. The petroleum spirit and other paraffin oils, by virtue of their diffuse solvent, stimulant, and antiseptic actions, are valuable both for external and internal use, the petroleum benzene or petroleum spirit being used for many of the purposes of oil of turpentine. Dr. M. M. Griffith asserts that the crude semisolid petroleum as it accumulates on the casings about oil wells is an invaluable remedy in chronic bronchitis and incipient phthisis. Huile de Cabian is a similar product which has long been famous in France as a remedy in lung diseases. Liquid petroleum is much used as a soothing local application in inflammation of the mucous membrane of the nose, throat, larynx, and even bronchial tubes.

(To be continued.)

